# Immune Checkpoint Inhibitors as Independent and Synergistic Drivers of SJS/TEN

**DOI:** 10.1001/jamaoncol.2025.4349

**Published:** 2025-10-30

**Authors:** Eric Milan Mukherjee, Dodie Park, Amir Asiaee, Matthew S. Krantz, Cosby A. Stone, Michelle Martin-Pozo, Elizabeth Phillips

**Affiliations:** 1 Department of Dermatology, Vanderbilt University Medical Center, Nashville, Tennessee; 2 Center for Drug Safety and Immunology, Vanderbilt University Medical Center, Nashville, Tennessee; 3 Department of Biostatistics, Vanderbilt University Medical Center, Nashville, Tennessee; 4 Department of Medicine, Division of Infectious Diseases, Vanderbilt University Medical Center, Nashville, Tennessee; 5 Institute for Immunology and Infectious Diseases, Murdoch University, Perth, Western Australia, Australia

## Abstract

This cross-sectional study examines the association of immune checkpoint inhibitor use with Stevens-Johnson syndrome, toxic epidermal necrolysis.

Immune checkpoint inhibitors (ICIs) are paradigm-shifting cancer treatments that are increasingly associated with Stevens-Johnson syndrome, toxic epidermal necrolysis (SJS/TEN) and other life-threatening cutaneous reactions. Differentiating ICI-induced true SJS/TEN from SJS/TEN-like reactions is difficult, the latter of which may be distinct lichenoid or bullous reactions.^1,2,3^ In some cases, ICI-related SJS/TEN-like reactions occur in association with human leukocyte antigen (HLA)–restricted drug culprits like allopurinol, suggesting a 2-hit mechanism.^4^ With increasing ICI use, a clearer understanding of their role in SJS/TEN is critical.

## Methods

We analyzed 13 986 839 deduplicated Food and Drug Administration Adverse Event Reporting System (FAERS) reports (2013-2023), containing 17 495 patients with SJS/TEN. We assessed the impact of ICI using logistic regressions adjusted for age, sex, cancer, polypharmacy, and strong (lamotrigine, trimethoprim-sulfamethoxazole, phenytoin, allopurinol, carbamazepine) or weak (azithromycin, clarithromycin, erythromycin, ciprofloxacin, levofloxacin, moxifloxacin, and acyclovir) culprit exposure.

To assess latency patterns, we performed Cox proportional hazards analyses among patients with SJS/TEN with documented latency. In 1 model, we compared latency between ICI-attributed and non–ICI-attributed cases, classifying primary suspect (PS) by ICI mechanism. In another, we used time-dependent Cox regression with interval splitting to dynamically update exposure to programmed cell death 1 (PD-1) and its ligand (PD-L1), cytotoxic T-lymphocyte antigen 4 (CTLA-4), or lymphocyte-activation gene 3 (LAG-3) inhibitors. Both models incorporated the same covariates as the logistic regression. Additional eMethods are in Supplement 1.

## Results

In a multivariable logistic regression (Table), ICI exposure was associated with increased risk of SJS/TEN (adjusted odds ratio [aOR], 9.14; 95% CI, 8.42-9.93; *P* < .001). Strong culprit drugs were the strongest independent predictors of SJS/TEN (aOR, 14.31; 95% CI, 13.77-14.87). Importantly, cancer diagnosis was inversely associated with SJS/TEN risk (aOR, 0.60; 95% CI, 0.58-0.63). Interaction terms revealed additive synergy between ICI exposure and culprit drugs. The ICI–strong culprit interaction yielded an attributable proportion (AP) of 0.38, indicating that 38% of the risk in co-exposed patients was attributable to interaction. For ICI-weak culprits, AP was even higher (0.52).

**Table.** Multivariable Logistic Regression Identifying Independent and Synergistic Predictors of Stevens-Johnson Syndrome, Toxic Epidermal Necrolysis (SJS/TEN)^a^

Variable	Total, No.	Patients with SJS-TEN, No.	aOR (95% CI)	*P* value
Independent covariates				
Age				
Spline term 1	NA	NA	.31 (.29-.33)	<.001
Spline term 2	NA	NA	.63 (.57-.70)	<.001
Spline term 3	NA	NA	.29 (.23-.37)	<.001
Spline term 4	NA	NA	.44 (.29-.68)	<.001
Sex				
Female	7 388 660	8591	1 [Reference]	NA
Male	4 863 436	6572	1.01 (.98-1.04)	.60
Not specified	1 734 743	2332	2.46 (2.33-2.59)	<.001
ICI				
No	13 797 009	16 525	1 [Reference]	NA
Yes	189 830	970	9.14 (8.42-9.93)	<.001
Strong culprit				
No	13 645 127	12 255	1 [Reference]	NA
Yes	341 712	5240	14.31 (13.77-14.87)	<.001
Weak culprit				
No	13 637 635	14 953	1 [Reference]	NA
Yes	349 204	2542	3.69 (3.51-3.88)	<.001
Cancer flag				
No	11 698 458	14 357	1 [Reference])	
Yes	2 288 381	3138	0.60 (0.58-0.63)	<.001
No. of drugs	Per unit increase		0.99 (0.98-0.99)	<.001
Age missing (imputed)				
Observed	7 844 874	13 922	1 [Reference]	NA
Imputed	6 141 965	3573	0.29 (0.27-0.30)	<.001
Interaction terms				
Term				
ICI strong culprit (multiplicative aOR)			0.28 (0.22-0.35)	<.001
RERI			13.69 (6.54-22.66)	
AP			0.38 (0.22-0.50)	
Synergy Index			1.64 (1.31-2.06)	
ICI weak culprit (multiplicative aOR)			0.73 (0.57-0.95)	.02
RERI			12.92 (7.36-20.14)	
AP			0.52 (0.38-0.63)	
Synergy Index			2.19 (1.68-2.86)	

Abbreviations: aOR, adjusted odds ratio; AP, attributable proportion; ICI, immune checkpoint inhibitor; RERI, relative excess risk due to interaction.

^a^ Adjusted odds ratios with 95% CIs are shown for independent covariates (age, sex, ICI use, strong and weak culprit drugs, cancer diagnosis, and polypharmacy) and for interaction terms between ICIs and culprit drug classes. Age was modeled using natural splines with 4 degrees of freedom. Additive interaction was quantified using the RERI, attributable proportion (AP), and synergy index. Notably, ICIs had a strong independent association with SJS/TEN (aOR, 9.14) and exhibited substantial synergy with both strong and weak culprit drugs (RERI, 13.69 and 12.92, respectively).

Patients with an anti–PD-1 as PS had a time to event of 27 days, compared with 13 days for non-ICI, 15 days for PD-L1, and 20 days for CTLA-4/PD-1 combination. We further evaluated latency patterns using 2 Cox models (Figure). In the time-dependent model, in which a time-dependent ICI exposure was considered, PD-1 inhibitors were associated with delayed SJS/TEN onset (hazard ratio [HR], 0.82; 95% CI, 0.72-0.94; *P* = .004). In the mechanism-based model, in which ICI or combinations coded as PS were compared, PD-1 inhibitors again exhibited delayed onset compared with non-ICI causative drugs (HR 0.71; 95% CI, 0.60-0.94; *P* < .001). In both models, cancer diagnoses was associated with later onset (HR 0.78; mechanism based: 95% CI, 0.72-0.84; for time-dependent: 95% CI, 0.71-0.84; *P* < .001). The direction of covariate associations were consistent between models.

**Figure.**
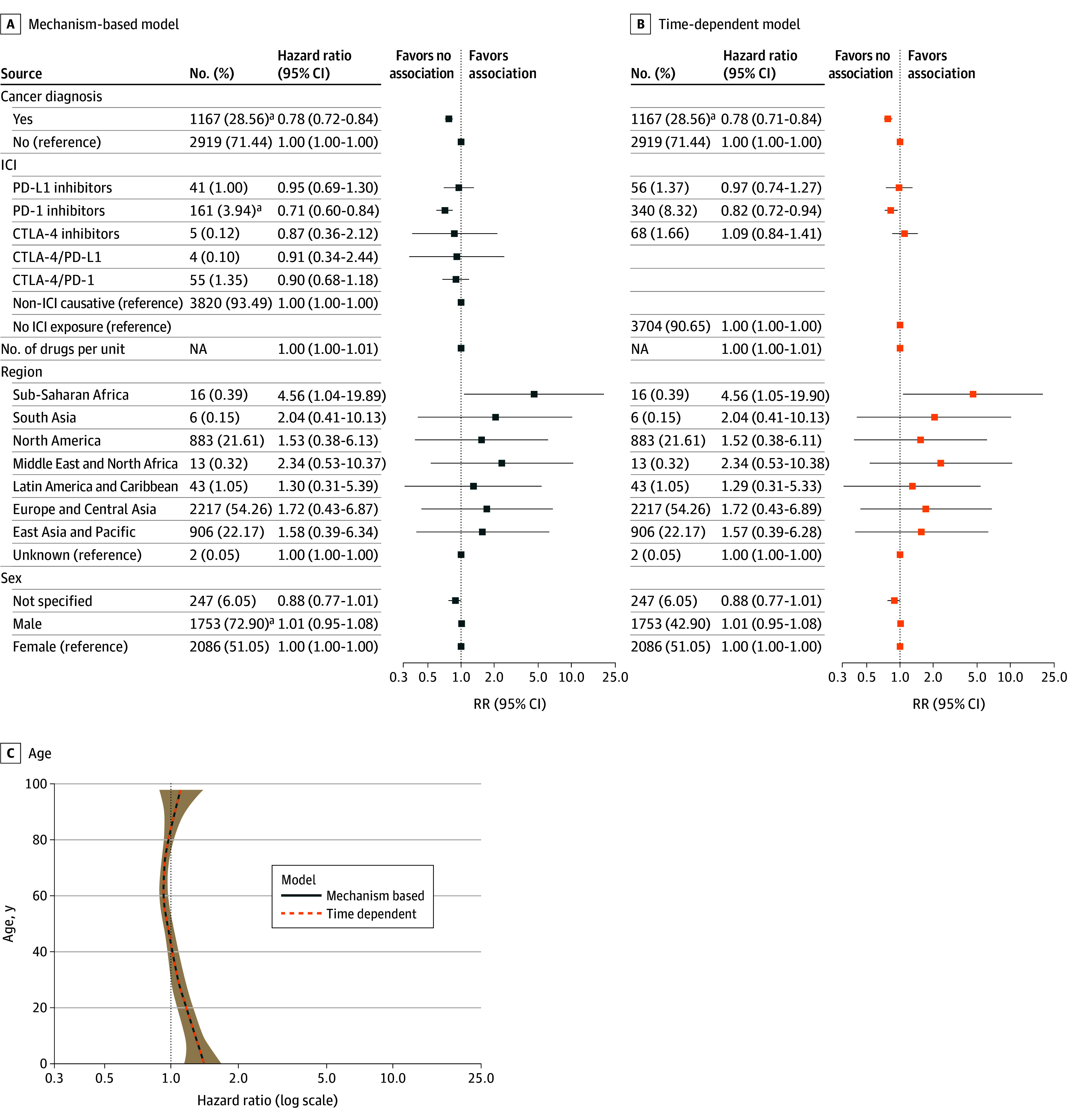
Latency Patterns of Stevens-Johnson Syndrome, Toxic Epidermal Necrolysis (SJS/TEN) Stratified by Immune Checkpoint Inhibitor (ICI) Exposure and Mechanism of Association Hazard ratios (HRs) for time to onset of SJS/TEN were estimated using 2 Cox models. A, a mechanism-based model considering cases in which ICIs were coded as primary suspect, divided by mechanism. B, a time-dependent model incorporating ICI exposure as a dynamic covariate. Both models identified programmed cell death 1 (PD-1) inhibition and cancer diagnosis as significantly associated with delayed onset. C, the effect of age using a spline term, revealing a nonlinear relationship between age and time to onset of SJS/TEN. Results across both models were consistent, supporting associations between ICI mechanism and latency patterns. ^a^*P* < .001.

## Discussion

Our findings confirm that ICIs were independently associated with increased risk of SJS/TEN and may synergize with high-risk small molecules to further amplify this risk. Both strong culprits (eg, allopurinol, trimethoprim-sulfamethoxazole) and weaker culprits (eg, fluoroquinolones, macrolides) were significant predictors of SJS/TEN, but their effects were substantially magnified in the presence of ICI exposure, supporting a model of additive or supra-additive risk. Furthermore, latency analyses revealed that ICI-associated SJS/TEN presents later than non-ICI cases, with anti–PD-1 therapies showing an onset period nearly 2-fold longer. These latency effects were consistent across both time-dependent and causative-agent Cox models.

This analysis is subject to the inherent limitations of spontaneous reporting systems such as FAERS, including underreporting, reporting bias, missing data, and the inability to confirm causality.

Together, these results suggest a 2-hit model, in which ICIs lower threshold for drug-specific T-cell activation. This model is supported by emerging mechanistic evidence on HLA-restricted, T-cell–mediated hypersensitivity, showing that ICIs can decrease threshold for T-cell activation.^5^ The delayed onset observed with ICI-associated SJS/TEN may further contribute to misattribution, increasing the risk of underrecognition or misdiagnosis in oncology settings. Our findings underscore the importance of careful coprescribing in patients treated with ICIs. These insights also reinforce the need for prospective studies and pharmacogenomic investigations to identify patients at highest risk and to develop guidelines for safer prescribing in cancer immunotherapy.

## Notes

Supplement 1. eMethods

Supplement 2. Data Sharing Statement
