# Supplementary material for: Immune Checkpoint Inhibitors as Independent and Synergistic Drivers of SJS/TEN
Source: JAMA Oncol. 2025 Oct 30;11(12):1542–5. doi: 10.1001/jamaoncol.2025.4349 (PMC13383115; doi:10.1001/jamaoncol.2025.4349)
Supplement: Supplement 1. — eMethods [file jamaoncol-e254349-s001.pdf]

Supplemental Online Content

Milan Mukherjee E, Park D, Asiaee A, et al. Immune checkpoint inhibitors as independent and synergistic drivers of SJS/TEN: an analysis of FAERS. *JAMA Oncol*. Published online October 30, 2025. doi:10.1001/jamaoncol.2025.4349

eMethods.

This supplemental material has been provided by the authors to give readers additional information about their work.

© 2025 Milan Mukherjee E et al. *JAMA Oncol*.

Data Preparation

We analyzed FAERS reports from 2013 to 2023 using a deduplicated dataset constructed as previously described.[1] We restricted analyses to the post-ICI era (2013 onward) and excluded duplicate reports, following standard pharmacovigilance practices.

Drugs coded as primary suspect (PS) were considered causative for the sake of this analysis. TMP-SMX exposure was comprehensively captured by merging reports listing trimethoprim, sulfamethoxazole, or pre-combined formulations. Exposure variables were constructed for immune checkpoint inhibitors (ICIs) when present in the drug list for a patient in any role. ICIs were grouped by mechanistic class (PD-1, PD-L1, CTLA-4, LAG-3), and additional variables included polypharmacy (number of unique drugs), sex, age, and cancer diagnosis (assigned using both indication keywords and manual curation of cancer-specific medications).

Age was missing in 43.9% of entries. To preserve modeling power while respecting variable distribution, we imputed missing ages using sequential hot deck imputation via the impute_shd() function from R's simputation package (v0.2.9), using the ten most age-correlated variables as donors. This approach preserves covariate relationships without assuming distributional form.

For time-to-event analyses, we calculated latency (TTE, in days) as the interval between drug initiation and SJS/TEN symptom onset, using FDA-submitted dates. We retained events with full month, day, and year information for both primary suspect drug and event, with plausible latency values between 1 and 180 days. In total, 4,086 SJS/TEN cases had usable TTE data and were included in survival analyses.

© 2025 Milan Mukherjee E et al. *JAMA Oncol.*

Logistic Regression Analysis

To assess whether immune checkpoint inhibitor (ICI) exposure modifies the association between small-molecule drugs and risk of SJS/TEN, we conducted a multivariable logistic regression that included interaction terms between ICI exposure and two drug classes: strong culprits (e.g., allopurinol, TMP-SMX) and weak culprits (e.g., macrolides, fluoroquinolones). The final model was specified as:

$$\text{logit}\left(P(\text{SJS/TEN})\right) = \beta_0 + \beta_1 \cdot \text{ICI} + \beta_2 \cdot \text{StrongCulprit} + \beta_3 \cdot (\text{ICI} \times \text{StrongCulprit}) + \beta_4 \cdot \text{WeakCulprit} + \beta_5 \cdot (\text{ICI} \times \text{WeakCulprit}) + f(\text{age}) + \beta_6 \cdot \text{sex} + \beta_7 \cdot \text{cancer} + \beta_8 \cdot \text{NumDrugs} + \beta_9 \cdot \text{ageMissing}$$

Where:

- f(age) is modeled using a natural spline with four degrees of freedom to capture nonlinear age effects.
- ageMissing is a binary variable indicating imputed vs. observed age, included to account for missing age values (imputed using sequential hot-deck imputation).
- Additional covariates included sex, cancer diagnosis, and polypharmacy (number of drugs).

We estimated multiplicative interaction effects for both ICI × Strong Culprit and ICI × Weak Culprit using interaction terms in the model. To assess additive interaction, we used the interactionR package to compute[2]:

- RERI (Relative Excess Risk due to Interaction)

  $$\text{RERI} = RR_{11} - RR_{10} - RR_{01} + 1$$

  where RR_{ij} denotes risk ratios for combinations of ICI (i) and culprit exposure (j).
- AP (Attributable Proportion due to interaction)

  $$\text{AP} = \frac{\text{RERI}}{RR_{11}}$$
- S (Synergy Index)

  $$\text{S} = \frac{RR_{11} - 1}{(RR_{10} - 1) + (RR_{01} - 1)}$$

© 2025 Milan Mukherjee E et al. *JAMA Oncol.*

These metrics quantify the degree of risk amplification due to combined ICI and culprit drug exposure, beyond the sum of their individual effects. Confidence intervals for RERI, AP, and S were derived using delta method standard errors as implemented in interactionR.

Time-to-Event Analyses

We performed two complementary Cox proportional hazards models to assess latency patterns in SJS/TEN cases, using documented time-to-onset (TTE) from drug initiation to symptom onset.

Model 1: Time-Dependent ICI Exposure

To address immortal time bias and examine mechanistic differences across immune checkpoint inhibitor (ICI) classes, we implemented a time-dependent Cox regression using interval-split data. Each patient's follow-up time was divided into risk intervals corresponding to exposure status for PD-1, PD-L1, CTLA-4, and LAG-3 inhibitors before the event date. For those cases with only month or year information, admin date was coded as the beginning of the month or the year (Jan 1), respectively. Time-dependent binary indicators were updated dynamically at the start of each interval to reflect ICI administration timing to avoid immortal time bias. The model was fit using the following structure:

$$\lambda(t) = \lambda_0(t) \exp\left(\beta_1 \cdot \text{PD-1}_t + \beta_2 \cdot \text{PD-L1}_t + \beta_3 \cdot \text{CTLA-4}_t + \beta_4 \cdot \text{LAG-3}_t + f(\text{age}) + \boldsymbol{\beta} \cdot X\right)$$

Where:

- $\lambda(t)$: Hazard function at time t
- $\lambda_0(t)$: Baseline hazard at time t
- $\text{PD-1}_t, \text{PD-L1}_t, \text{CTLA-4}_t, \text{LAG-3}_t$: Time-dependent ICI exposure indicators
- $f(\text{age})$: Penalized spline function for age (4 degrees of freedom)
- $\boldsymbol{\beta} \cdot X$: Linear predictor for covariates (e.g., sex, cancer status, region, number of drugs)

© 2025 Milan Mukherjee E et al. *JAMA Oncol.*

This approach allowed for estimation of hazard ratios specific to ICI subclasses and identification of delayed versus early-onset risk patterns associated with different immunotherapy agents.

Model 2: ICI vs Non-ICI Culprit Comparison

To compare latency distributions between ICI-attributed and non-ICI-attributed SJS/TEN cases, we restricted analysis to reports with clearly identified primary suspect drugs. We classified each causative agent as an ICI or non-ICI and further subtyped ICIs by mechanism (PD-1, PD-L1, CTLA-4, or combinations thereof). A standard Cox proportional hazards model was then constructed using TTE as the outcome:

λ(t)=λ0(t)ex (β·Mechanism+f(age)+β·X)\lambda(t) = \lambda_0(t) \exp\left(\beta \cdot \text{Mechanism} + f(\text{age}) + \beta \cdot X\right)

As above, covariates included penalized splines for age, along with sex, cancer status, polypharmacy, and region. Mechanism of action (e.g., PD-1, PD-L1, CTLA-4, CTLA-4/PD-1) was treated as a categorical variable with “non-ICI” as the reference group. ICIs administered in combination were collapsed into a single composite variable using the earliest shared start date and unique mechanism combinations (e.g., “CTLA-4 / PD-1”).

Visualization and Interpretation

For Cox models, hazard ratios with 95% CI were extracted from each model and visualized via forest plots. Age effects were displayed separately using spline-predicted hazard functions, plotted against binned age values with ribbons representing ±1.96 standard error. Spline terms captured nonlinear associations between age and latency. All models were implemented in R using the survival (v3.7-0), pspline (1.0-20), splines (v4.4.2), and broom (v1.0.7) R packages.

References

1. Mukherjee EM, Park D, Krantz MS, Stone CA, Martin-Pozo M, Phillips E. Demographics, Latency, and Mortality of Severe Cutaneous Adverse Reactions in an FDA Database. Published online May 16, 2025:2025.03.05.25323441. doi:10.1101/2025.03.05.25323441

2. Alli BY. InteractionR: An R package for full reporting of effect modification and interaction. *Software Impacts*. 2021;10:100147. doi:10.1016/j.simpa.2021.100147

© 2025 Milan Mukherjee E et al. *JAMA Oncol.*
