# Supplementary material for: Immune Checkpoint Inhibitors as Independent and Synergistic Drivers of SJS/TEN
Source: JAMA Oncol. 2025 Oct 30;11(12):1542–5. doi: 10.1001/jamaoncol.2025.4349 (PMC13383115; doi:10.1001/jamaoncol.2025.4349)
Supplement: Supplement 2. — Data Sharing Statement [file jamaoncol-e254349-s002.pdf]

Data Sharing Statement

Data
Data available: Yes
Data types: Deidentified participant data
How to access data: Data and methods are available at https://github.com/capuhcheeno/SCARs_ICI-Manuscript-Scripts/tree/main/ICI%20and%20Culprit%20Analysis.
When available: With publication

Supporting Documents
Document types: Statistical/analytic code
How to access documents: https://github.com/capuhcheeno/SCARs_ICI-Manuscript-Scripts/tree/main/ICI%20and%20Culprit%20Analysis.
When available: With publication

Additional Information
Who can access the data: Anyone requesting the data (the data is based on FAERS, which is publically available)
Types of analyses: For any purpose (the data is based on FAERS, which is publically available)
Mechanisms of data availability: With investigator support
